# MRI after Whoops procedure: diagnostic value for residual sarcoma and predictive value for an incomplete second resection

**DOI:** 10.1007/s00256-021-03790-z

**Published:** 2021-04-26

**Authors:** Mohammed H. A. Alramdan, Ömer Kasalak, Lukas B. Been, Albert J. H. Suurmeijer, Derya Yakar, Thomas C. Kwee

**Affiliations:** 1 Department of Radiology, Nuclear Medicine and Molecular Imaging, Medical Imaging Center, University of Groningen, University Medical Center Groningen, Hanzeplein 1, P.O. Box 30.001, 9700 RB Groningen, The Netherlands; 2 Department of Surgical Oncology, University of Groningen, University Medical Center Groningen, Groningen, The Netherlands; 3 Department of Pathology, University of Groningen, University Medical Center Groningen, Groningen, The Netherlands

**Keywords:** Magnetic resonance imaging, Repeat surgery, Sarcoma

## Abstract

**Objective:**

To determine the value of MRI for the detection and assessment of the anatomic extent of residual sarcoma after a Whoops procedure (unplanned sarcoma resection) and its utility for the prediction of an incomplete second resection.

**Materials and methods:**

This study included consecutive patients who underwent a Whoops procedure, successively followed by gadolinium chelate-enhanced MRI and second surgery at a tertiary care sarcoma center.

**Results:**

Twenty-six patients were included, of whom 19 with residual tumor at the second surgery and 8 with an incomplete second resection (R1: *n* = 6 and R2: *n* = 2). Interobserver agreement for residual tumor at MRI after a Whoops procedure was perfect (*κ* value: 1.000). MRI achieved a sensitivity of 47.4% (9/19), a specificity of 100% (7/7), a positive predictive value of 100% (9/9), and a negative predictive value of 70.0% (7/17) for the detection of residual tumor. MRI correctly classified 2 of 19 residual sarcomas as deep-seated (i.e., extending beyond the superficial muscle fascia) but failed to correctly classify 3 of 19 residual sarcomas as deep-seated. There were no significant associations between MRI findings (presence of residual tumor, maximum tumor diameter, anatomic tumor extent, tumor margins, tumor spiculae, and tumor tail on the superficial fascia) with an incomplete (R1 or R2) second resection.

**Conclusion:**

Gadolinium chelate-enhanced MRI is a reproducible method to rule in residual sarcoma, but it is insufficiently accurate to rule out and assess the anatomic extent or residual sarcoma after a Whoops procedure. Furthermore, MRI has no utility in predicting an incomplete second resection.

## Introduction

Superficial soft-tissue sarcomas are malignant mesenchymal tumors located within the cutaneous and/or subcutaneous layers [1]. Ideally, magnetic resonance imaging (MRI) should be performed for local staging and surgical planning, followed by percutaneous (image-guided) biopsy to confirm the diagnosis of sarcoma, before embarking on any surgical procedure [1, 2]. However, in patients presenting with a superficial soft-tissue lesion, the differentiation between sarcoma and a benign entity can be challenging based on history taking and clinical examination [3, 4]. Furthermore, benign superficial soft-tissue lesions greatly outnumber superficial soft-tissue sarcomas [3, 4]. It has been estimated that only 1 out of 100 primary care consultations regarding new soft tissue masses are malignant [3, 5]. Because of these epidemiological data, many physicians simply do not include the possibility of sarcoma in their differential diagnosis [4]. Consequently, there are patients who inadvertently undergo a non-oncological resection for a presumed benign superficial soft-tissue lesion that proves to be a sarcoma upon pathological examination. An unplanned sarcoma resection (whether located in the superficial soft tissues of elsewhere) is called a Whoops procedure.

In patients who underwent a Whoops procedure, second surgery with tumor bed excision is recommended, with the goal of obtaining complete tumor removal with an appropriately wide margin of resection, ideally performed in a specialized sarcoma center [6]. Prior to the second surgery, MRI can be performed for operative planning. However, the value of MRI for the detection of residual disease remains unclear, with varying diagnostic values that were reported in the recent literature [7–10]. In addition, it is currently unknown how often MRI upgrades a presumed superficial soft-tissue sarcoma to a deep-seated soft-tissue sarcoma (i.e., extending beyond the superficial muscle fascia). This information is important to the surgeon to determine the extent of the second resection. Furthermore, the value of MRI in predicting the technical outcome of the second surgery in terms of achieving a complete pathological (R0) resection [11] is currently unknown. When a complete tumor resection is not achieved with the second surgery, additional local treatment, either by means of radiation therapy and/or a third surgery, is usually deemed necessary. If MRI can identify patients in whom the second surgery is likely going to fail, this information may be helpful to the surgeon to plan a more extensive second resection beforehand.

The purpose of this study was therefore to determine the value of MRI for the detection and assessment of the anatomic extent of residual sarcoma after a Whoops procedure and its utility for the prediction of an incomplete second resection.

## Materials and methods

### Study design

This retrospective study was approved by the local institutional review board of the University Medical Center Groningen, and the requirement for informed consent was waived. All consecutive patients who underwent a Whoops procedure between August 2010 and January 2020, and who were referred to our tertiary care sarcoma center, were potentially eligible for inclusion. Patients were excluded if no MRI was performed between the Whoops procedure and the second surgery, if no second surgery was performed, or if radiation therapy was given between the MRI examination and the second surgery.

### MRI acquisition

MRI was performed to detect any residual disease after the Whoops procedure and to plan the extent of excision of the second surgery. Different clinical 1.5-T and 3.0-T MRI systems were used for image acquisition. All MRI protocols included at least T1-weighted, (fat-suppressed) T2-weighted, and gadolinium chelate-enhanced sequences (including subtraction gadolinium chelate-enhanced images, generated by digitally subtracting the pre-contrast from the post-contrast sequences), with a slice thickness ranging between 1 and 5 mm and oriented in at least two perpendicular directions.

### MRI evaluation

**Fig. 1:**
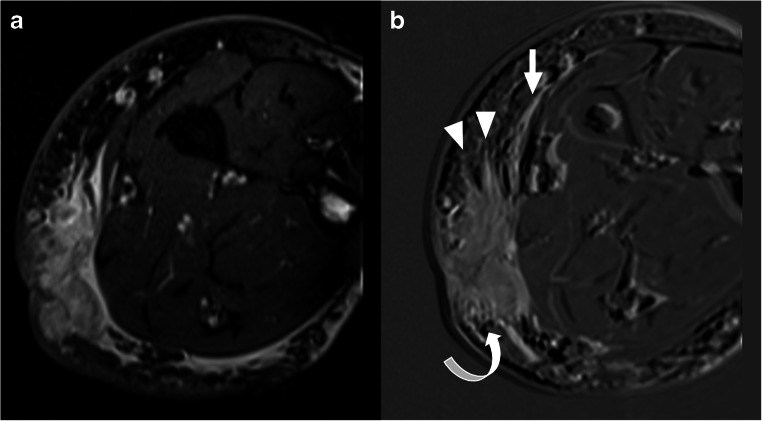
Axial T2-weighted (**a**) and subtraction gadolinium chelate-enhanced (**b**) images at the level of a forearm demonstrate a residual tumor (pathologically proven myxofibrosarcoma) after a Whoops procedure with unsharp tumor margins (curved arrow), tumor spiculae (arrowheads), and a tumor tail on the superficial muscle fascia (straight arrow)

MRI scans were independently evaluated by two musculoskeletal radiologists (T.C.K. and Ö.K.), both with more than 5 years of experience in the evaluation of sarcomas. These two readers were blinded to all surgical and pathological findings and to each other’s findings. The probability of residual tumor (which was considered present when MRI demonstrated a contrast-enhancing nodular lesion or mass) was assessed on a scale from 1 to 5 (1 = very unlikely, 2 = unlikely, 3 = indeterminate, 4 = likely, 5 = very likely). If residual tumor was considered present at MRI (i.e., likelihood scores of 4 or 5), the maximum diameter of the tumor (in any plane) was measured. In addition, anatomic tumor extent was classified as superficial (i.e., no extension beyond the superficial muscle fascia), as extension beyond the superficial muscle fascia without muscle (and/or tendon) involvement, or as extension beyond the superficial muscle fascia with muscle (and/or tendon) involvement. Tumor margins (sharp or unsharp), tumor spiculae (present or absent), and tumor tail on the superficial fascia (present or absent) were also determined on gadolinium chelate-enhanced sequences (Fig. 1). T2-weighted imaging does not add value over gadolinium chelate-enhanced sequences for tumor detection, and T2-weighted imaging is rather non-specific to delineate tumor boundaries in this setting, in the authors’ experience. Therefore, only gadolinium chelate-enhanced sequences were used for residual sarcoma evaluation. The maximum tumor diameters measured by the first radiologist were used for all further analyses (except for the interobserver agreement analysis). All other disagreements in MRI assessments between the two radiologists were resolved in a consensus meeting.

### Second surgery

All second surgeries were done by oncological surgeons (who completed general surgery training and subspecialized in sarcoma surgery) in our tertiary care sarcoma center, after discussing each case in a multidisciplinary meeting with representatives from the departments of oncologic surgery, radiology, pathology, radiation therapy, and plastic surgery.

### Pathological examination

All resected specimens obtained at the time of the Whoops procedure and the second surgery were evaluated by a pathologist (A.J.H.S.) with more than 30 years of experience in the evaluation of sarcomas. Tumors were classified according to the 2020 World Health Organization Classification of Tumours of Soft Tissue and Bone [12]. The presence and anatomic extent (including histological invasion with respect to the superficial muscle fascia) of residual tumor in the tissue resected at the second surgery were determined. The presence of residual tumor in the surgical bed after the second surgery was also assessed, using the residual tumor (R) classification (with R0 corresponding to complete resection, R1 corresponding to microscopic residual tumor, and R2 corresponding to macroscopic residual tumor [11]).

### Statistical analysis

The interobserver agreement regarding the assessment of residual tumor, anatomic tumor extent, tumor margins, tumor spiculae, and tumor tail on the superficial fascia at MRI was analyzed using (weighted) *κ* statistics (with *κ* < 0.2 indicating poor agreement, *κ* > 0.2 to *κ* ≤ 0.4 indicating fair agreement, *κ* > 0.4 to *κ* ≤ 0.6 indicating moderate agreement, *κ* > 0.6 to *κ* ≤ 0.8 indicating substantial agreement, and *κ* > 0.8 to *κ* ≤ 1 indicating almost perfect agreement). A Bland-Altman analysis was performed to determine the interobserver agreement regarding the measurement of the maximum tumor diameter at MRI (mean bias ± limits of agreement). The sensitivity, specificity, positive predictive value (PPV), and negative predictive value (NPV) of MRI for the detection of residual disease after a Whoops procedure were assessed, using pathologic findings as reference standard. Agreement between MRI and pathological findings regarding the anatomic extent of residual tumor was descriptively analyzed. Finally, logistic regression analyses were performed to determine the association between several MRI findings (presence of residual tumor, maximum tumor diameter, anatomic tumor extent, tumor margins, tumor spiculae, and tumor tail on the superficial fascia) with an incomplete (R1 or R2) second resection. *P* values less than 0.05 were considered statistically significant. Statistical analyses were executed using the MedCalc version 17.2 Software (MedCalc).

## Results

### Patients

**Fig. 2:**
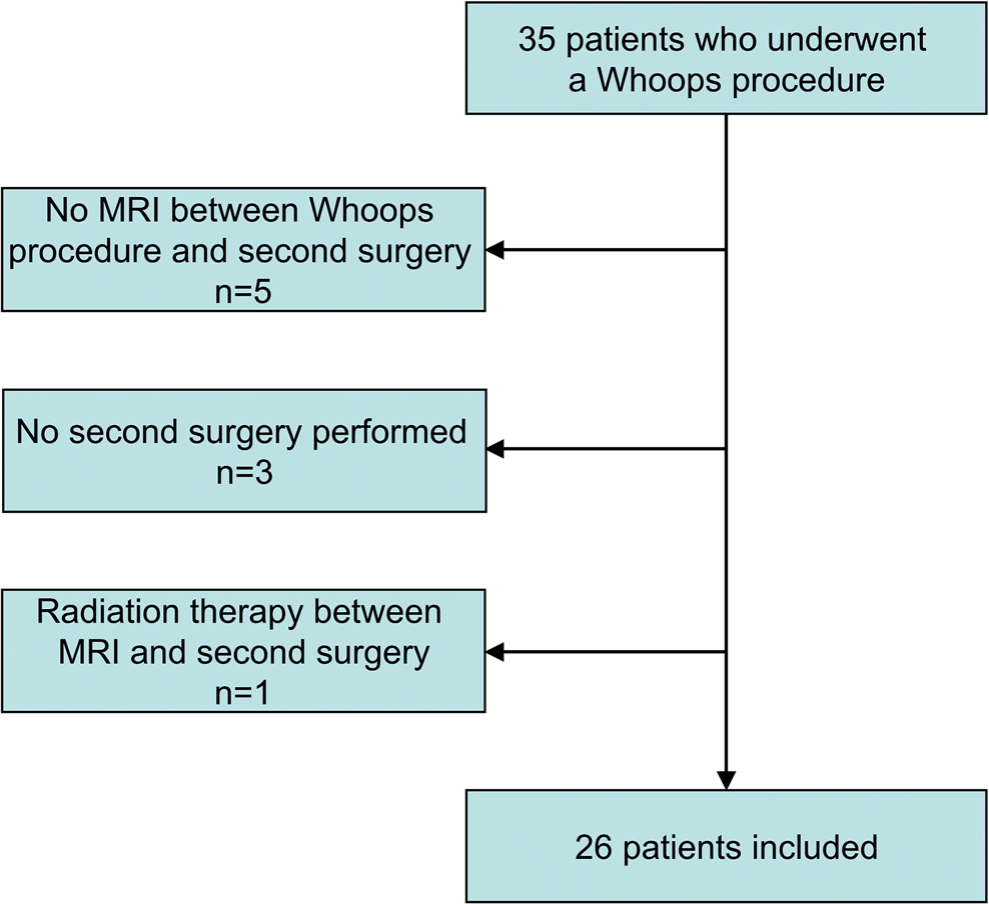
Flowchart of patient inclusion

A total of 35 consecutive patients who underwent a Whoops procedure between August 2010 and January 2020, and who were referred to our tertiary care sarcoma center, were potentially eligible for inclusion. Eventually, 26 patients (19 men and 7 women) with a median age of 64.5 years (range: 21–98 years) were included (Fig. 2). The types of sarcomas are shown in Table 1. Sarcomas were located in the leg (*n* = 7), arm (*n* = 6), back (*n* = 5), chest wall (*n* = 4), abdominal wall (*n* = 2), and gluteal area (*n* = 2). Five of these 26 patients underwent additional radiation therapy before MRI and the second surgery. None of the patients had any signs of metastatic disease after clinical and imaging work-up (including chest CT) at initial presentation after the Whoops procedure. MRI included pre- and post-gadolinium-enhanced non-fat-suppressed T1-weighted images in 18 patients and pre- and post-gadolinium-enhanced non-fat-suppressed and fat-suppressed Dixon-based T1-weighted images in 8 patients.

**Table 1:** Types of sarcoma that were included

Type of sarcoma	No.
Myxofibrosarcoma	8
Leiomyosarcoma	5
Pleomorphic sarcoma	4
Synovial sarcoma	4
Dermatofibrosarcoma	1
Liposarcoma	1
Myofibroblastic sarcoma	1
Extraskeletal osteosarcoma	1
Rhabdomyosarcoma	1

### MRI interobserver agreement

Interobserver agreement for residual tumor at MRI was perfect (*κ* value of 1.000). Interobserver agreement for anatomic tumor extent, tumor margins, tumor spiculae, and tumor tail on the superficial fascia at MRI ranged from substantial to perfect (*κ* values of 0.780 to 1.000) (Table 2). Mean bias ± limits of agreement was 3 mm ± 15 mm for maximum tumor diameter measurements at MRI.

**Table 2:** Interobserver agreement at MRI

MRI finding	*κ* value	95% confidence interval
Residual tumor	1.000	–
Anatomic tumor extent	1.000	–
Tumor margins	0.835	0.523–1.000
Tumor spiculae	0.780	0.367–1.000
Tumor tail on the superficial fascia	1.000	–

### Value of MRI for residual tumor detection and assessment of anatomic tumor extent

**Fig. 3:**
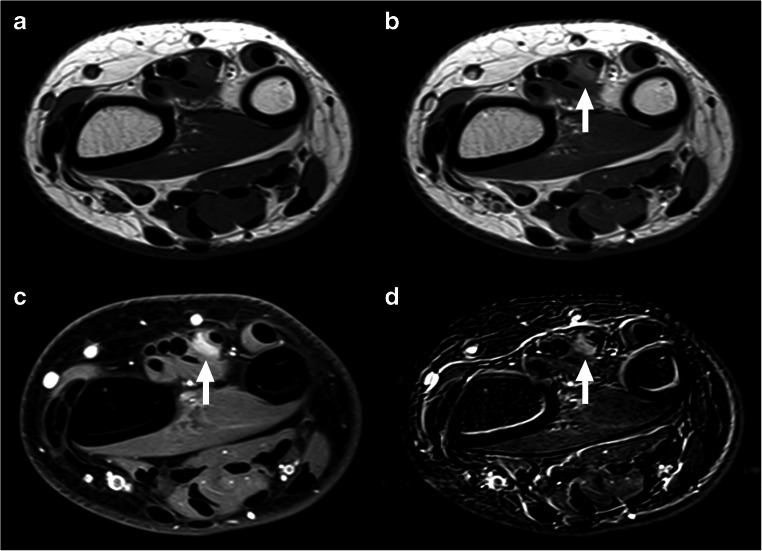
Example of true-positive MRI scan in a 35-year-old woman with synovial sarcoma who had undergone a Whoops procedure at the dorsal side of the right wrist. Axial T1-weighted (**a**), gadolinium chelate-enhanced T1-weighted (**b**), fat-suppressed gadolinium chelate-enhanced T1-weighted (**c**), and subtraction gadolinium chelate-enhanced (**d**) images at the level of the right wrist. There is a contrast-enhancing nodular lesion adjacent to the extensor digiti minimi tendon (arrows), which was interpreted as deep-seated residual tumor, and confirmed by pathological examination after second surgery

**Fig. 4:**
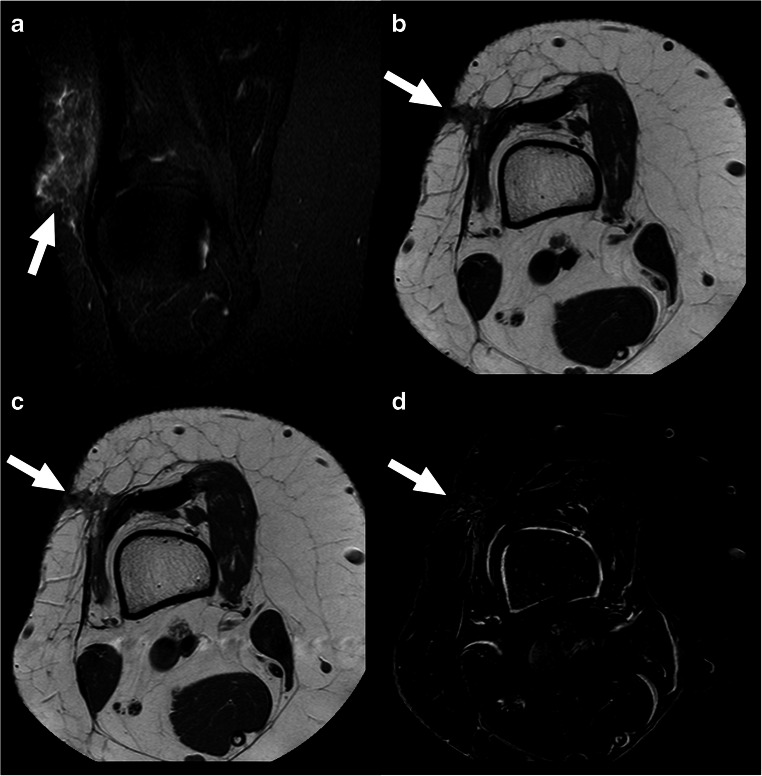
Example of false-negative MRI scan in a 21-year-old woman with synovial sarcoma who had undergone a Whoops procedure at the lateral side of the distal upper right leg. Coronal fat-suppressed T2-weighted (**a**), axial T1-weighted (**b**), gadolinium chelate-enhanced T1-weighted (**c**), and subtraction gadolinium chelate-enhanced (**d**) images at the level of the of the distal right upper leg. There are superficial soft-tissue changes at the lateral side of the distal upper right leg (arrows), without a contrast-enhancing nodular lesion or mass, which was interpreted as postsurgical changes without residual tumor. However, pathological examination after second surgery showed residual synovial sarcoma, extending beyond the superficial muscle fascia with muscle involvement

MRI achieved a sensitivity of 47.4% (9/19), a specificity of 100% (7/7), a PPV of 100% (9/9), and an NPV of 70.0% (7/17) for the detection of residual tumor after a Whoops procedure. Figures 3 and 4 show examples of true-positive and false-negative MRI scans. MRI suggested 5 residual sarcomas with a deep-seated location (i.e., extending beyond the superficial muscle fascia) and 4 residual sarcomas with a superficial location. Pathologic examination demonstrated 5 residual sarcomas with a deep-seated location and 14 residual sarcomas with a superficial location. MRI correctly classified 2 of 19 residual sarcomas as deep-seated but failed to correctly classify 3 of 19 residual sarcomas as deep-seated (Table 3).

**Table 3:** Anatomic tumor extent according to MRI and pathology

Tumor location at MRI	Tumor location at pathology	No.
Superficial (*n* = 5)	Superficial	4
	Muscle involvement	1
Beyond superficial muscle fascia (*n* = 2)	Superficial	1
	Beyond superficial muscle fascia	1
Muscle involvement (*n* = 2)	Superficial	1
	Muscle involvement	1
No tumor detected (*n* = 17)	Superficial	8
	Beyond superficial muscle fascia	1
	Muscle involvement	1
	No tumor detected	7

### Value of MRI for the prediction of an incomplete second resection

Eight of 19 cases with residual tumor at the second surgery had an incomplete second resection (R1: *n* = 6 and R2: *n* = 2). There were no significant associations between any of the MRI findings (presence of residual tumor, maximum diameter of the tumor in any plane, anatomic tumor extent, tumor margins, tumor spiculae, and tumor tail on the superficial fascia) with an incomplete second resection (i.e., R1 or R2 resection) (Table 4). These results did not change when excluding the 5 cases who had additional radiation therapy before MRI and the second surgery (Table 5).

**Table 4:** Univariate regression analysis on the association between MRI findings and an incomplete second resection (R1 or R2)

MRI finding	Odds ratio	95% CI*	*P* value
Residual tumor	2.600	0.462–14.631	0.277
Maximum tumor diameter	1.019	0.986–1.053	0.251
Anatomic tumor extent	1.600	0.670–3.818	0.287
Tumor margins	0.714	0.063–8.151	0.782
Tumor spiculae	1.143	0.088–14.777	0.919
Tumor tail on the superficial fascia	3.000	0.452–19.929	0.257

*Confidence interval

**Table 5:** Univariate regression analysis on the association between MRI variables and an incomplete second resection (R1 or R2) excluding patients who had undergone radiation therapy before MRI and second surgery

MRI finding	Odds ratio	95% CI*	*P* value
Residual tumor	2.000	0.241–16.613	0.526
Maximum tumor diameter	1.017	0.954–1.084	0.613
Anatomic tumor extent	2.048	0.686–6.115	0.186
Tumor margins	1.300	0.095–17.717	0.846
Tumor spiculae	1.300	0.095–17.717	0.846
Tumor tail on the superficial fascia	1.300	0.095–17.717	0.846

*Confidence interval

## Discussion

The results of this study show that MRI (gadolinium chelate-enhanced imaging) is very specific in the detection of residual sarcoma after a Whoops procedure but that its sensitivity is too poor to exclude residual disease. Although it can be speculated that close follow-up MRI may be a potential alternative to second surgery in patients without any visible tumor at MRI, the risk of developing (lung) metastatic disease during this “watch and wait” period may be considered unacceptably high. Therefore, MRI cannot be used to select patients who can be spared a second surgery. Second, of the 19 residual sarcomas, 5 were deep-seated of which only two were recognized as being deep-seated on MRI examination. Therefore, MRI cannot be reliably used to plan the appropriate extent of the second resection. The inability to accurately delineate the anatomic extent of residual sarcoma before the second surgery is also reflected by the fact that 42.1% of patients with a residual sarcoma underwent an incomplete second resection. Third, although it was hypothesized that several tumor characteristics at MRI (size, anatomic extent, margins, spiculae, and tail on the superficial fascia) would increase the risk of an incomplete resection, our results did not indicate such a relationship. The main limitation of MRI, similar to that of other currently available clinical imaging techniques such as ultrasonography, CT, and PET, is its inability to detect microscopic disease. New imaging techniques are required to improve operative planning and the success of the second surgery after a Whoops procedure. This clinical need may perhaps be met by using targeted, fluorescently labeled ligands combined with optical imaging instruments with micron resolution to guide surgical resection, as demonstrated by some preliminary studies [13–15].

**Fig. 5:**
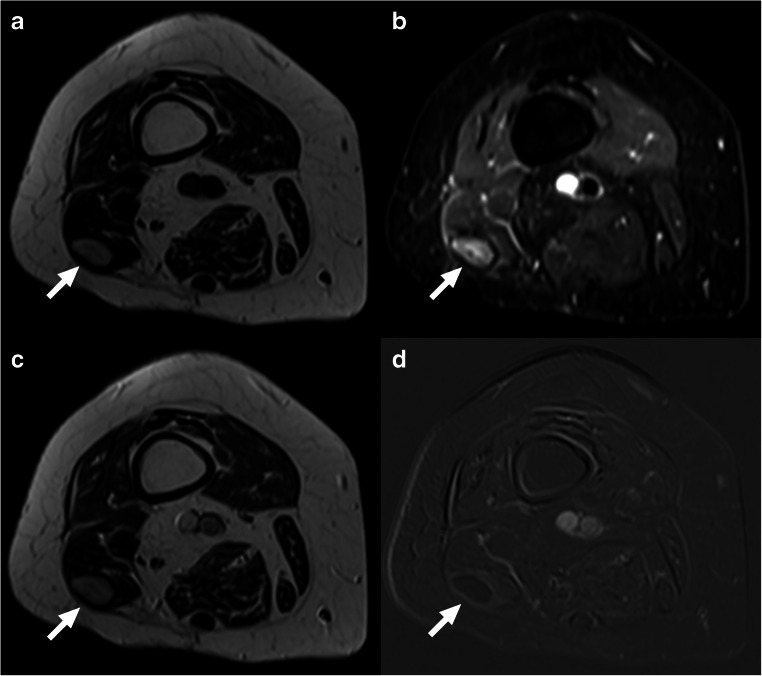
Example of a subacute hematoma with high T1 and T2 signal intensity (not to be confused with residual tumor) in a 77-year-old woman with pleomorphic sarcoma who had undergone a Whoops procedure at the lateral side of the distal right upper leg. Axial T1-weighted (**a**), fat-suppressed T2-weighted (**b**), gadolinium chelate-enhanced T1-weighted (**c**), and subtraction (**a** subtracted from **c**) gadolinium chelate-enhanced (**d**) images at the level of the distal right upper leg. There is a lesion with high T1 and T2 signal intensity in the distal biceps femoris muscle (deep-seated location) and a surrounding low signal intensity rim (**a** and **b**, arrows). The lesion also has a high signal on the gadolinium chelate-enhanced T1-weighted image (**c**, arrow), and it remains unclear if the lesion is enhancing. However, the subtraction gadolinium chelate-enhanced image clearly shows no nodular or mass-like enhancement (**d**, arrow), which favors subacute hematoma and makes residual tumor unlikely. Pathological examination after second surgery showed hematoma and no residual tumor

There are a few recent studies that investigated the value of MRI for the detection of residual tumor after a Whoops procedure. Patkar et al. [7] included 55 patients and reported MRI to have a sensitivity of 86.7% and a specificity of 90.9% when using “a focal mass typically of high signal on T2-weighted and short-time inversion recovery sequences” as criterion for residual disease. In a study by Gingrich et al. [8] in 64 patients, MRI was reported to achieve a sensitivity of 57.8% and a specificity of 89.5% when using “focal or discrete enhancing mass” as criterion for residual tumor. In another study by Wang et al. [9] in 20 patients, MRI was found to have a sensitivity of 36.4% and a specificity of 80.0% when using “nodular or mass-like enhancement” as criterion for residual disease. Finally, Erol et al. [10] enrolled 50 patients and reported MRI to have a sensitivity of 79.4% and a specificity of 82.4%, without mentioning any criteria for residual tumor at MRI. Unlike these previous studies [7–10], the present study did not have any false-positive among a total of 26 MRI scans. Although Gingrich et al. [8] and Wang et al. [9] used the same MRI criterion for residual tumor as the present study (i.e., a contrast-enhancing nodular lesion or mass), they still had 2 false-positives among 64 MRI scans and 2 false-positives among 20 MRI scans, respectively. The false-positives in the studies by Gingrich et al. [8] and Wang et al. [9] may possibly be attributed to subacute hematomas with high T1 signal intensity on post-contrast sequences that were mistaken for residual tumors, as also shown in Fig. 2b in the study by Wang et al. [9]. To more accurately differentiate subacute hematoma from residual tumor, and to identify true enhancement, we propose to use subtraction gadolinium chelate-enhanced images, which are generated by digitally subtracting the pre-contrast from the post-contrast sequences (note that these pre-contrast and post-contrast sequences should be acquired with the same parameters for this purpose) (Fig. 5). Otherwise, the findings of recent studies on this topic are in line with that of the present study, in that a negative MRI does not reliably exclude the presence of residual disease. However, previous studies neither investigated the value of MRI for assessing the anatomic extent of residual tumor, nor its utility for predicting the pathological success of the second surgery.

The present study had some limitations. First, this study had a retrospective design, and the sample size was relatively small. Nevertheless, the consecutively enrolled cases are representative of the clinical patient spectrum that is seen in a specialized sarcoma center. Second, the value of advanced MRI techniques such as perfusion or diffusion-weighted imaging was not investigated. However, although some studies reported that these advanced MRI techniques may have value in the detection and localization of soft-tissue sarcomas [16–19], they still have a limited spatial resolution and therefore cannot detect microscopic residual tumor. Third, this study did not irrefutably prove that MRI after a Whoops procedure has no influence on the success of the second surgery. This can only be investigated by a randomized trial. Moreover, a gross estimation of residual tumor location and size, when visible at MRI, can still be considered helpful to guide surgery. Therefore, we do not advocate to omit MRI before the second surgery but to optimize its use and interpretation and to be aware of its limitations in this setting. Fourth, at present, the surgeons at our institution mainly use the MRI scan to obtain a gross anatomic overview of where residual sarcoma is located and how deep it reaches. However, the resection itself is primarily based on macroscopic intraoperative findings, and not on the area of abnormal signal on MRI. This may have affected the correlation between MRI findings and the outcome of the second surgery. The use of intraoperative MRI may perhaps improve the success rate of the second surgery, which is a topic for further research.

In conclusion, gadolinium chelate-enhanced MRI is a reproducible method to rule in residual sarcoma, but it is insufficiently accurate to rule out and assess the anatomic extent or residual sarcoma after a Whoops procedure. Furthermore, MRI has no utility in predicting an incomplete second resection.
